# Development and Application of SAW Filter

**DOI:** 10.3390/mi13050656

**Published:** 2022-04-20

**Authors:** Pu Chen, Guangxi Li, Zhiyuan Zhu

**Affiliations:** 1 Henan Province Engineering Research Center of Ultrasonic Technology Application, Pingdingshan University, Pingdingshan 467000, China; cp120010@163.com; 2 College of Electronic and Information Engineering, Southwest University, Chongqing 400715, China

**Keywords:** component, SAW, filter, RF, 5G

## Abstract

With the in-depth advancement of the fifth generation (5G) mobile communication technology, the technical requirements for filters are also constantly improving. Surface acoustic wave (SAW) filters are widely used in home TV, mobile communications, radio frequency filters and radar due to their simple structure, few mask layers, easy miniaturization, and low cost. Through the continuous improvement of communication technology, SAW has developed into various high-performance acoustic filters from bulk SAW with the support of some new architectures, new materials and advanced modeling techniques. This paper analyzes and reviews the research situation of SAW filter technology.

## 1. Introduction

The rapid development of wireless communication applications has put forward urgent requirements for broad band and high-speed data transmission. From a technical and economic point of view, mobile communication is a key element in realizing a platform in the form of social innovation services and user applications. The overall demand for mobile broad band data services is growing, mobile traffic has been experimenting with exponential growth every year, and will continue to rise in the future driven by ubiquitous demand. In the field of mobile communication, the fifth generation (5G) technology is a new technology after 4G. Compared with 3G and 4G, the transmission rate and data carrying capacity of 5G network have been greatly improved, the utilization rate of spectrum resources is high, and the communication protocol is complex. To meet the bandwidth needs, more new frequency bands have been released for mobile communications, and today, more than 30 frequency bands are used, and this number is increasing, or has been liberated from other applications such as analog TV, and dedicated to this A new purpose. In addition, more efficient transmission standards such as LTE (Long Term Evolution) and LTE-A (Long Term Evolution-Advanced) have been adopted [1]. Acoustic filtering is attractive because typical acoustic wavelengths are about five orders of magnitude smaller than electromagnetic wavelengths at the same frequency, making submillimeter-sized resonators or even complete filters suitable for mobile applications possible. The exact width of the strips that make up the IDT and the exact metal thickness of the IDT both affect the performance and frequency stability of the filter as well as the bandwidth, which is required for high lateral precision. Availability of a portfolio of filters and duplexers to easily create highly integrated modules combining filter solutions for a wide variety of frequency bands and regions, minimum size requirements, and customer preferences.

For nearly 40 years, surface acoustic wave devices have been key components of wireless data transmission systems. It was first widely used in intermediate frequency (IF) filters of television receivers. Because the frequency bands are so close together, the filter’s passband skirt must be very steep to ensure sufficient stopband attenuation in adjacent frequency bands. Micro-acoustics works by exploiting a variety of different propagation velocities: the technology is highly relevant in the radio frequency (RF) field, ranging from tens of megahertz to several gigahertz. In this case, at the system TEM level, duplexers and multiplexers are key elements. The number of service and roaming frequency bands around the world continues to grow, and RF filters have been rapidly developed to better meet the growing demand [2]. On the other hand, the minimization of filter footprint is also a very important task in filter design while improving simultaneous filtering performance. This puts more emphasis on the latest developments in size and advanced LTE carriers, carrier aggregation (which allows data transmission to perform tasks on multiple frequency bands simultaneously) [3].The emergence of LTE-Carrier Aggregation (CA) as a means of increasing data rates in mobile device communications has resulted in a paradigm shift in acoustic filter design due to the eager acceptance of users, the need to stream HD video at high data rates and other innovations. The q-factors of lumped-unit lc filters and transmission line filter designs are often not high enough to replace acoustic filters, but they are still used with acoustic filters. For example, most antenna-side multiplexers, which separate low, mid, and high frequency bands, are partially or fully based on LC filter designs. Low loss, high isolation, high linearity multiplexing capability will continue to be a differentiating factor in this market. To be successful, it is absolutely essential that filtering technology allows and supports these needs [4]. The market for discrete filter components is getting smaller as the RF front-end of mobile phones becomes more complex, which includes more and more RF filters/duplexers to allow mobile phones to operate in different frequency bands. Integrating RF filters/duplexers in RF front-end modules is standard in today’s smartphones. RFSAW filters use two different filtering techniques. One is the so-called DMS filter technique, which is a tandem structure of input and output transducers with the addition of a reflective grating on the outside of the acoustic structure. The second filtering technique is the trapezoidal filtering technique [5].

In the future, the required steep passband skirts can only be achieved by interconnecting filters composed of high-q single-port resonators, but the fabrication tolerances and temperature sensitivity of the devices will have to be reduced. So further challenges are to reduce filter passband ripple, reduce passband attenuation, reduce non-linearity [6] (which is necessary because CA brings many hybrid products into the usable band), whatever the new filter looks like, must help to increase the effective data rate and functional density of digital communication systems. This paper analyzes and reviews the research situation of SAW filter technology.

## 2. SAW

Research on SAW dates back to 1885, when Lord Rayleigh predicted its mode of transmission and propertiest [7,8]. In 1965, R.M. White and F.W. volmer invented the Interdigital Transducer (IDT) [9]. The SAW on the piezoelectric single crystal substrate can be well excited by IDT, and surface acoustic wave (SAW) filtering technology has gradually attracted people’s attention since then. The basic IDT consists of a three-port structure consisting of two acoustic ports and one electrical port, usually the transducer metal is chosen for chemical inertness or acoustic matching with piezoelectric materials [10]. The working mechanism of SAW is that when alternating current is applied to the input IDT, a periodic electric field will be generated [11]. When the period of the IDT is close to the wavelength of the SAW, it will resonate and generate a SAW of a certain frequency. Subsequently, the SAW propagates along the piezoelectric substrate, passing through Delay to reach another IDT output terminal, and convert the sound wave signal into an electrical signal output through the piezoelectric effect [12]. The orientation of the crystal, the thickness of the piezoelectric material and the geometry of the metal transducer determine the type of sound waves produced [12]. Different types of sound waves are used to achieve different filtering effects required.

**Figure 1:**
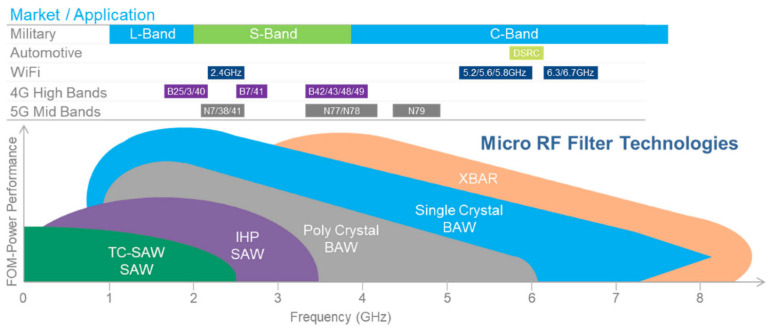
Market Application and Frequency Band Allocation of RF Filtering Technology [2].

The current success of SAW filters is attributed to their small size, low insertion loss (IL), and superior out-of-band rejection [13]. However, with the rapid increase in the complexity of the communication network, the number of RF circuits in the RF front-end of the mobile phone also increases at the same time. Current BAW (Bulk Acoustic Wave) devices, such as thin film bulk acoustic resonators (FBARs) are also widely used in RF front-ends because of their higher power supply durability and lower losses, however multimode RF BAW filters are difficult to mass-produce because Tricky mechanisms require coupling tuning, which is an extremely difficult process. SAWs are widely used as receive filters and as the receiving part of duplexers, allowing us to arrange multiple resonant locations by appropriate, known as dual-mode SAW (DMS) filters, by combining multiple single-mode resonators, and setting Appropriate resonant frequency and setting of clamping capacitors have the possibility of realizing multimode filters [14]. But at the same time, with the addition of carrier aggregation technology, SAW duplexer and other technologies, the requirements for the linearity specifications of the device are becoming more and more strict, and the nonlinearity and severe distortion of the SAW device will reduce the signal quality at the front end of the radio frequency. Min, etc., have seriously hindered its development, so it is necessary to continuously solve the factors that make the filter non-linear, such as the influence of temperature and power on it. After solving these problems, SAW will go further in the communication RF front end [15].The application and frequency band allocation of RF filtering technology in the current market are shown in Figure 1.

## 3. SAW Filter Technology

### 3.1. Bulk SAW

Bulk SAW devices have been around for more than 30 years, are low cost and are easy to manufacture. However, bulk SAW devices have poor power handling and temperature coefficient of frequency (TCF) and relatively poor Q.

TV-IF (television intermediate frequency) SAW filters began mass production in the mid-70 s [16] Surface acoustic wave resonators change the relative size and elastic properties of the substrate due to external mechanical vibrations, which changes the acoustic wave propagation speed of the device [17]. In 1992, Morita et al. proposed a low-loss filter in the literature, which can achieve wide bandwidth in different LiTaO_3_ tangential directions. This filter technology is realized by using two different longitudinal acoustic modes in a coupled filter structure, which is composed of Consists of an inline structure and an IDT backed by two reflectors. DMS filter has very special function, it can realize balun function and impedance transformation [18]. The typical frequency response of a DMS filter is shoulder behavior in the higher stopband and excellent far-end out-of-band rejection [19]. At the 1998 IEEE International Symposium on Frequency Control, Kinsmann presented an overview of crystal filters, including trapezoidal and lattice structures, in his invited paper [20].

One of the low-cost bandpass filter architectures is a ladder topology, which consists of multiple replicas of SAW resonators of series and parallel type. The filter set based on this structure is limited due to layout and materials used, so that the acoustic resonator cannot have any electromechanical coupling coefficient. Most SAW-based achievable bandwidth (BW) RF filters are limited by electromechanical coupling by the coefficient k_2_t of the piezoelectric substrate. For example, based on conventional acoustic wave resonator filter design techniques (lattice and ladder-type SAW filter design), the achievable 3 dB fractional bandwidth (FBW) is only 0.4 and 0.8 times that of the SAW resonator k_2_t. In 2020, Ting Cai, put a lot of effort into increasing the bandwidth of filters based on SAW resonators, to achieve bandwidth that is not constrained by the electromechanical coupling coefficients they employ. A novel dual narrowband BPF design is based on a hybrid resonator of transmission line TFS and high-Q SAW, the achievable bandwidth of the filter is not constrained by the electromechanical coupling coefficients it adopts, the SAW resonator and its transmission characteristics, the filter can be flexible control [21]. In the same year, Runqi Zhang, proposed a hybrid low-cost bandpass filter. This new filter topology is based on a two-path coupling scheme. Unlike traditional SAW-based low-cost bandpass filters, it adopts a single SAW The resonator BVD model can effectively reduce the cost of the design, optimization and manufacturing process; and the design has been constructed and tested with two BPF prototypes centered at 418 MHZ. The measured results are close to the predicted results [22].

Currently in SAW resonators and filters in RF front-end modules for mobile communications, LiNbO_3_ and LiTaO_3_ are still the most widely used materials [23]. However, the operating frequencies of most SAW devices based on these traditional piezoelectric materials are below 3 GHZ, and in many applications, SAWs, if limited in the GHZ frequency range, will result in an inability to meet the growing demand for high operating frequencies from advanced mobile communications. system frequency. The speed of sound in the substrates is usually fixed, setting the gap width between the substrates is very complicated, unlike the case of conventional SAW filters (with 0.5 metallization ratio), the excitation of surface acoustic waves in the GHZ range on GaAs, its SAW retardation The operating frequency of the wire does not decrease the fabrication pitch size, but increases by adopting the IDT geometry, which has never been adopted on GaAs, 2017, Silvia Rizzato, Marco Scigliuzzo, by acting on the IDT between the finger width and the pitch Metallization ratios, without the use of expensive sbmicron/nanofabrication techniques, demonstrate the excitation of surface acoustic waves in the GHZ range on GaAs and facilitate the generation of higher harmonic modes [24]. In 2018, Qi Li, Su-Lei Fu, found that when using Ni as the substrate, the thickness of Ni will directly affect the Al interdigital transducer, and found that when the thickness is 6 nm, the Al(111) texture of the Ni substrate is The structure is the strongest, the surface is the flattest, and the electromigration test lifetime is nearly 10 times longer than that of the Al(111) texture without Ni substrate. The power durability of the 1.5 GHZ SAW filter with Al thin film added on 6 nm Ni substrate is also improved by 29.0~32.5 dB compared with the 1.5 GHZ SAW filter without Ni substrate added [13].

### 3.2. TC-SAW

When the ambient temperature changes, acoustic devices typically experience a frequency shift that increases approximately linearly with temperature. It can be characterized by the temperature coefficient of frequency (TCF) [25], which is usually in the range of −30 to −45 ppm/K for LiTaO_3_ [26], although today’s SAW filter technology is very mature, but in RF Driven by front-end market demand, it has continued to develop in recent years. Temperature stability is characterized by the temperature coefficient of frequency (TCF), which varies as a fraction of the temperature T at a particular frequency [27].

(1) $$\mathrm{TCF}\;=\frac{1}{f}\;\frac{\partial f}{\partial T}=\;\mathrm{TCV}-\mathrm{CTE},$$

(2) $$\mathrm{TCV}\;=\frac{1}{V}\;\frac{\partial V}{\partial T}$$

With the increasing communication requirements, the standard surface acoustic wave (SAW) filter has a high temperature drift frequency, which produces nonlinear frequency changes to temperature changes, which seriously affects the filter performance and cannot meet certain strict requirements [28]. specifications, so BAW filters including thin-film bulk acoustic resonators and solid-mounted resonator filters have been developed to meet this requirement. However, BAW filters are generally more expensive and larger than SAW filters [29]. TC-SAW (Temperature compensated SAW) is widely used in mobile phone RF front-end modules today due to its low TCF, low insertion loss, high rejection level, and high roll rate. The current TC-SAW has two commonly used structures [1]: One is an attached silicon dioxide (SiO_2_) structure on lithium niobate deposited on the inter-digital sensor (IDT) on a substrate [30]. Commonly used materials such as LN and LT become soft under the action of T, and the TCF is negative. Since SiO_2_ has no obvious temperature change, the TCF is positive. In this way, the absolute value of the overall (TCF) is reduced. However, since the amorphous SiO_2_ is non-piezoelectric [31], the infiltration will also lead to a decrease in the electromechanical coupling coefficient of the propagating SAW, and the deposition of SiO_2_ will also affect the reflection characteristics of the surface acoustic wave. The other is a lithium tantalate (LT) structure on sapphire/Si/other materials [32], Sapphire itself has good temperature compensation and a large Young’s modulus. It is very difficult to manufacture and requires careful control of electrode thickness and oxide film thickness.

The current temperature compensation technology is generally divided into two types 1. Silica wrap (SiO_2_ capping layer is deposited on a high voltage substrate. IDT(s) is placed on the boundary between the two) [33,34,35,36,37] 2. Wafer bonding technology (A thin piezoelectric plate is bonded to another thick substrate) [27,38]. Japan’s Murata and Panasonic companies use these two technologies. Murata uses SiO_2_ with flat top surface, Panasonic uses SiO_2_ and convex top surface, and Taiyo Yuden uses room temperature wafer bonding technology to provide frequency temperature compensation for SAW devices, so that the absolute value of TCF of the device decreases [27].

#### 3.2.1. SiO_2_ Overlay

In 2005, Michio KADOTA, by combining a flat silicon dioxide film and a relatively thick LiTaO_3_ substrate [39], first developed large reflection coefficient, optimal electromechanical coupling coefficient and good mechanical resonance Q value suitable for US-PCS duplex [40,41,42]. In 2007, Michio et al. used Love wave to combine flat SiO_2_ film, high-density metal (Cu) electrode and YX-LiNbO_3_ substrate to fabricate a small surface acoustic wave duplexer, and obtained good resonance Q value, TCF and The best electromechanical coupling coefficient [43]. Hiroyuki, in 2008, fabricated a miniature SAW duplexer using a SiO_2_ capping layer/thick Al electrode/rotating y-cut LiNbO_3_ substrate structure [44]. In 2011, S. Matsuda, M. Hara observed that fluorine-doped silicon oxide (SiOF) has a good temperature elasticity coefficient by using Fourier transform infrared spectroscopy [45], and applied it in radio frequency SAW to compensate its temperature coefficient. With the increase of fluorine content r in silicon (SiOF), the negative TCF of LiNbO_3_ will increase, so that its absolute value decreases, and the quality factor does not change significantly. From the Fourier transform infrared spectrum, fluorine-doped silicon oxide (SiOF) has better temperature compensation ability than SiO_2_. In 2015, Yiliu Wang et al. adopted a piston-type modal sensor, which reduced the TCF by increasing the silicon oxide thickness, and designed a frequency band 13 duplexer, which was the first in the market to meet the NS07 suppression specification. 13 Band SAW Duplexer [46].

Nowadays, finite element analysis (FEM) can be used to simulate surface acoustic waves, but due to the limitation of calculation time and storage space, as well as the limitation of a large number of degrees of freedom. The finite element method is not suitable for 3D models to simulate actual SAW devices [25]. Julius Koskela proposed a two-dimensional algorithm hierarchical cascading technique (HCT) [47]. Through the periodic structure of the surface acoustic device, the device is divided into small areas, which can greatly reduce the calculation time and ensure the accuracy. On this basis, Marc Solal used HTC to simulate the full 3D surface wave resonator [48]. It is proved that this method can not only solve two-dimensional problems, but also allows to solve the whole three-dimensional problems, and the calculation time is also within a reasonable range. It can be applied to leaky surface acoustic wave resonators and temperature compensated surface acoustic wave resonators on lithium tantalate. Through further development in 2019, Xinyi Li et al. applied graphics processing unit (GPGPU) to HCT, which can significantly accelerate HCT, especially in large-scale scenes. In 2020, Naoto Matsuoka, studied the excessive scattering loss of SiO_2_/128-LN TC-SAW resonator on HCT finite element method. The simulation results show that the figure of merit decreases significantly with the increase of the number of acoustic scattering mechanisms, but the estimated value Q is still much higher than the actual value, indicating that acoustic scattering at structural discontinuities is not the main loss mechanism [49].

#### 3.2.2. Wafer Bonding

A thin piezoelectric plate is combined with another thick base substrate. If the thickness of the plate is greater than several SAW wavelengths, the plate through which the SAW propagates is equivalent to a semi-infinite plate. Bonding suppresses top surface thermal expansion for SAW propagation when the substrate is a rigid material and the CTE is small [27].

A “new” substrate for temperature compensated SAW ladder filters was published in 2003 by Kadota et al. [42]. In 2004, M. Miura used the combination of sapphire and lithium tantalate substrates in [23], which greatly suppressed the thermal expansion of LiTaO_3_. Due to the large thermal conductivity and small dielectric constant of sapphire, Miura et al. proposed the use of lithium tantalate (LiTaO_3_)/sapphire bonded substrate technology in a personal communication service (PCS) band 2 duplexer, suitable for 1.9 GHZ US-PCS band with sufficient power endurance and good temperature coefficient of frequency (TCF). In [50] in 2004, O. Kawachi, N. Taniguchi et al. used the same technique to develop an 8-band (900 MHZ) duplexer. Good insertion loss without spurious response, and good temperature coefficient over frequency are obtained. Recently, 2nd generation (2G) Global System for Mobile communications (GSM), Code Division Multiple Access (CDMA), 3rd generation (3G) W-CDMA (Wideband CDMA), 4th generation (4G) Long Term Evolution (Mobile communication systems such as LTE) show a trend of diversification [51]. In 2010, H, Kobayashi, K. Tohyama found that a hybrid substrate composed of a thin layer of LiTaO_3_ combined with a low thermal expansion coefficient support material could replace the traditional LiTaO_3_ bulk substrate to improve the temperature coefficient of frequency (TCF) of surface acoustic wave devices. Using a thinner LiTaO_3_ layer reduces the TCF significantly [25], but on the other hand, also results in a larger spurious response in the filter characteristics. The bottom of the LiTaO_3_ layer is rough enough that this problem can be effectively alleviated. Correct choice of support material can further suppress spurious responses. Bonding to LiTaO_3_ (LT) and LiNbO_3_ (LN) wafers using spinel by bonding and direct bonding techniques has also been studied [52]. 2021 R. Ruby designs a novel lithium lithium tantalate (LT) bonded to a silicon hybrid substrate (SiSAW) [29].

**Figure 2:**
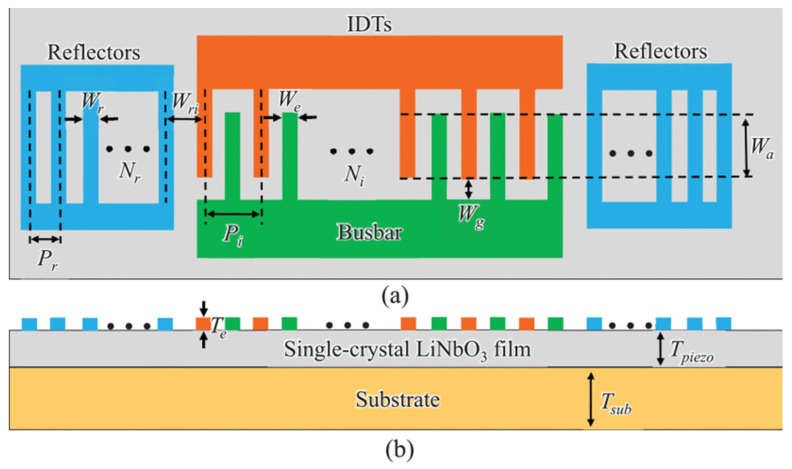
Schematic of a single-port resonator with key design parameters. (**a**) Top view. (**b**) Sectional view [53].

In 2020, Shibin Zhang prepared single-crystal X-cut LiNbO_3_ thin films on 4H-SiC substrates by ion cutting and wafer bonding processes [53]. The schematic diagram of the single-port resonator is shown in Figure 2 with a high electromechanical coupling coefficient of 26.9% and a high Q value of 1226, with a center frequency of 2.29 G, the insertion loss is 1.38 DB, and it has a low temperature coefficient. Although the power handling is limited by arc discharge and IDT, it still shows that the acoustic device platform on LiNbO_3_ on SiC has great potential in RF applications.

#### 3.2.3. Other Temperature Compensation Methods

**Figure 3:**
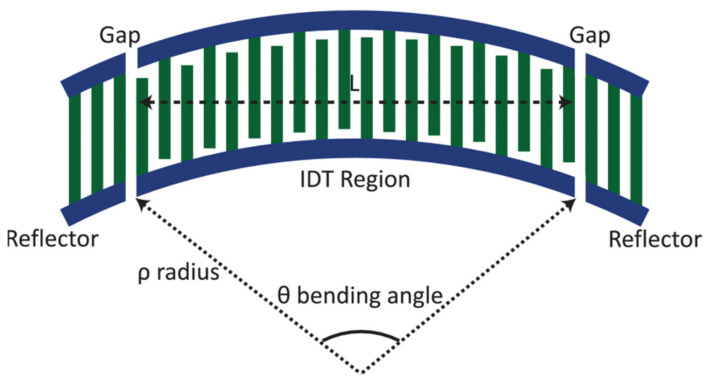
Bent TC-SAW Resonator [26]. © [2019] IEEE. Reprinted, with permission, from [26].

The fact that the higher TC-SAW wave modes in the resonator have larger losses at the resonator boundary. After bouncing multiple times along the length of the resonator, high wave modes leak energy from the resonator busbar. There have been many effective methods to reduce the number of transverse modes of the resonator to reduce the passband loss. For example, by bending the acoustic resonator waveguide, the conventional TC-SAW resonator, due to its lateral nature, has IDT-generated Rayleigh waves perpendicular to the busbars, generating lateral modes in the IDT region. When the boundary has curvature, the radiation loss is larger. The smaller the radius of curvature, the greater the radiation loss. The higher-order modes have a smaller inclination angle from the normal, closer to the critical angle, and have larger losses than the fundamental-order modes. In this case, in 2019, Yuhao Liu, Jiansong Liu found that when the surface acoustic wave resonator is bent, the fundamental mode can still be turned on, but due to the increase in radiation loss, the higher-order transverse mode will leak out [26]. The curved TC-SAW resonator is shown in Figure 3. The bending angle is defined as the length of the resonator IDT region divided by the radius. When the optimum curvature for the desired specific frequency is found, it is calculated and simulated without affecting the quality factor of the resonator. The method not only effectively reduces losses, but also has a simple design method and does not increase manufacturing difficulties.

In TC-SAW filters, Cu is widely used as an IDT electrode because Cu has a larger reflection coefficient than Al. However, unlike Al, Cu film is limited by strain energy and boundary. In 2021 Rongxuan Su, Sulei Fu proposed Ti(1)/Cu(1)/Ti(2)/Cu(2)/Ti(3) 5 Layered electrode structure [54] to improve the power durability of the TC-SAW filter. The results show that the IDT electrode can not only withstand a high power of 34.3 dBm, but also can increase the failure time to about 104 times that of the 3-layer electrode. Furthermore, with the help of finite element simulations and TEM characterizations, the behavior of TC-SAW filters at high powers is deeply investigated. The fine-grain strengthening of the bottom edge of the electrode improves the stress resistance of the electrode. Therefore, the use of 5-layer electrodes is a simple and effective method to improve the power durability of TC-SAW filters, which is suitable for mass production.

At present, the design of surface acoustic wave devices is entirely done through computer simulation, and the accuracy of which has a great influence on the achievable device performance. The modal coupling (COM) model has been widely used in acoustic simulation and its applicability has been widely recognized. In TC-SAW devices, lateral modes also cause stray resonances, and their suppression is also critical. The traditional COM model deals with one-dimensional surface acoustic waves, which is inaccurate for transverse mode simulation, while the two-dimensional COM model is computationally complex and time-consuming. The COM model is applied to the simulation of each transverse modal resonance, by accumulating all The response yields the overall characteristics of the device [39]. Simplified calculation time and cost. At the same time, the simulation accuracy is verified by experiments, and the transverse mode frequency and excitation replication obtained from the specific experiments can be correctly matched.

### 3.3. Hybrid Substrate SAW

Hybrid substrates with large impedance ratios (high Q), spurious free properties up to 14 GHZ, and almost 0 TCF (2 ppm K–1 at series resonance) [55,56,57,58].This technology is still in its infancy, the substrates are very expensive, At the same time, the resonant mode is accelerated (the frequency is increased), and the substrate linearity, resistivity, thermal conductivity [55], and CMOS (Complementary Metal-Oxide-Semiconductor) electronic compatibility are also the difficulties of this technology.

#### 3.3.1. IHP

**Figure 4:**
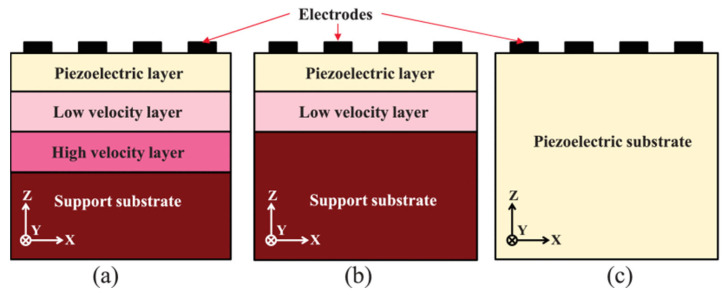
The basic structure of I.H.P (**a**) three-layer structure, (**b**) two-layer structure, (**c**) standard 42YX-LT substrate [64]. © [2019] IEEE. Reprinted, with permission, from [64].

In 2016, Tsutomu Takai et al. adopted a thin LT plate in [59] to propagate LSAW on a Si substrate, and demonstrated a figure of merit of 4000, a TCF of −8 ppm/°C, and this SAW resonator duplexer with low insertion loss and very narrow transition band. Known as IHP-SAW(Incredibly high performance SAW). In the same year, Tsutom Takai used IHP-SAW in [60] to design a high-performance Band4-Band25 multiplexer. Using these technologies, various multiplexers for other frequency bands (such as high frequency bands, etc.) can also be developed. In frequency division duplex (FDD) CA, the acoustic multiplexer is the most important key device, which can be improved by out-of-band reflection to allow the use of this IHP ladder filter for carrier aggregation. For higher frequencies above 3 GHZ, BAW filters are considered a better solution than SAW filters because of their increased electrode resistance. In 2017, Tsutomu Takai et al. adopted the single-port resonator of I.H.P. to fabricate SAW after optimizing the multilayer structure [61]. Experimental results show that the Bode-Q value successfully exceeds 6000 at 0.9 GHZ to over 1900 at 3.5 GHZ, which is more than 3 times higher than the conventional 42YX-LT SAW resonator. A very small TCF of −8 ppm/°C is also achieved, along with wider bandwidth and excellent thermal performance. Developed a new Wi-Fi filter with very narrow adjacent gap and a new Band 25/66/30 hexaplexer, one of the most difficult multiplexers, using I.H.P. In the same year, a new multilayer structure was developed using SiO_2_ as the low-resistance layer and AlN as the high-resistance layer under the thin LT layer. A single-port resonator was fabricated using the new substrate, and the experimental results showed that the developed resonator has a Bode-Q of over 4000 and a TCF of −8 ppm/°C, which are 4 times and 1/5 of the former, respectively. The same as the conventional 4° YX-LT SAW resonators, respectively. Using this technology, a 25-band duplexer with extremely narrow duplex gap has been successfully developed [62], which has extremely low insertion loss, steep cut-off characteristics and stable temperature characteristics. In 2018, Yuichi Takamine, Tsutomu Takai, a novel SAW device using a very thin piezoelectric layer with surface energy confinement effect and achieving ultra-high Q greater than or equal to BAW. 3.5 GHZ center frequency I.H.P. surface acoustic wave resonator, test performance shows low passband loss and good transition band steepness [63] Then in 2019, the triple-layer LT/SiO_2_/AlN structure was adopted in [64] to simplify the double-layer structure on the Si substrate. Three-layer structure, simplified structure, compared with standard 42YX-LT substrate, as shown in Figure 4.

The piezoelectric thin plate multilayer structure is called IHP SAW. When the acoustic wave propagates near the boundary of the low-velocity area and the high-velocity area, it will be confined in the low-velocity area. As shown in Figure 4a, the surface acoustic wave generated by the piezoelectric layer will be confined in the piezoelectric layer and the low-velocity layer, reducing the loss and thus Improved quality factor Q. On this basis, Tsutomu Takai reduced the number of layers from 3 to 2 layers, AIN and Si. The phase velocities of AIN and Si are not much different in SH-mode and both are much larger than the SiO_2_ phase velocity, so AIN is extracted. The propagation of SAW on the surface is analyzed by FEM (finite element method), and compared with the three-layer structure. The results show that in the double-layer structure, the acoustic energy of the surface acoustic wave is confined to the surface of the substrate, and still has a high Q value, and further calculated the correlation between thickness and cutting angle, and determined the optimal TCF and K2 values.

IHP/POI devices are hybrid SAW devices, bonded to silicon (or sapphire or quartz) using thick (>3 μm) LT. These devices show good TCF and power handling, with Q ranging from 1600 to 2300, and the advantage of silicon carrier wafers compared to sapphire or quartz is the heat extraction properties of silicon. Silicon conducts heat ~4 times better than sapphire and ~16 times better than quartz [29]. Excellent thermal extraction is the key to Tx filter applications.

#### 3.3.2. HAL

Heterogeneous acoustic layer (HAL) SAW device is a new type of SAW device [65], which adopts solid-mounted single crystal piezoelectric thin plate, and can obtain very high impedance ratio (ZR) and small temperature coefficient of frequency (TCF) at the same time [1].

In 2018, Michio Kadota and Yoshimi Yunoki reported a new type of hetero acoustic layer (HAL) surface acoustic wave (SAW) resonator with high impedance ratio and quality factor Q [66]. 2020 Michio Kadota, Yoshimi Ishii, in [67], prototyped parallel and series type band-stop filters at 0.9 GHZ using LT/quartz HAL SAW resonators. Due to its large impedance ratio in the stopband, it has a steep attenuation at the junction of the passband and stopband. In the passband frequency, the attenuation is very small, which will be used in carrier aggregation technology and IoT systems.

#### 3.3.3. LLSAW

Longitudinal leaky surface acoustic wave (LLSAW) mode has faster phase velocity and electromechanical coupling coefficient than Rayleigh wave, but its decay speed is much larger than other SAW modes on unimorphic piezoelectric substrates.

In 2013, Tetsuya Kimura and Katsuya Daimon designed 2.4 GHZ LLSAW in [68]. The substrate consists of a thin LiNbO_3_ plate and a SiO_2_/AlN reflector stacked on a glass substrate. Although the reflector suppresses energy leakage to a certain extent, but due to its low reflectivity, the final impedance ratio and bandwidth are 60 dB and 6.4%, respectively. In 2018, Tetsuya Kimura used platinum instead of AlN as the LLSAW resonator of the composite substrate reflector in X, which significantly improved the emissivity. In the case of 3.5 GHZ, the final experimental impedance and bandwidth were 71 dB and 9.5%, respectively [69]. Tetsuya Kimura, Masashi Omura 2018 Apply the inter-digital transducer cycle of 3.5 GHZ utilizing IHP SAW to another new SAW device LLSAW [70], and the results show that good transducer performance can still be obtained in the 5 GHZ range. In 2019, Masashi Suzuki and Naoya Sawada found that LLSAW propagating on a high-voltage electrical ScAlN layer/sapphire or quartz substrate also has high phase velocity, high K2, and low attenuation [71].

#### 3.3.4. Other Types

In 2000, Jun Tsutsumi formed a new type of reflection filter using SAW waveguide technology in [72], realizing a compact IF filter. In the second year, in 2001, Jun Tsutsumi studied the acoustic energy loss of the grating waveguide SAW filter. Based on the experiment, it was concluded that by increasing the thickness of the aluminum film, the insertion loss of the mirror filter using the SAW grating waveguide could be reduced by 2 dB [73]. For the layered surface acoustic wave device, it is not only affected by the substrate, but also affected by the interdigitated metal electrodes. In 2018, Qi Li, Sulei Fu, in [74] prepared 2.1 GHZ surface acoustic wave filters with Al/Ti/Cu/Ti four-layer electrodes with different Cu thicknesses. With Al (111 nm)/Ti (5 nm)/Cu (15 nm)/Ti (5 nm) electrodes, the power endurance was improved from 24.5 dBm to 28 dBm. In 2019, Qi Li and Sulei Fu prepared a series of 2.7 GHZ surface acoustic wave filters using Al/Cu/Ti three-layer electrodes. In the high-power load test, this Cu-doped α-Al and θ-CuAl_2_ structure can handle the stress of the finger well, so as to obtain higher anti-acoustic radiation performance, which provides a kind of high-power surface acoustic wave filter. promising solution [75].

In [76] 2020 studied the design of SH-SAW resonators based on LiNbO_3_/SiO_2_/Si functional substrates. It is proposed that the multi-frequency SH-SAW resonator can be realized based on the LiNbO_3_/SiO_2_/Si wafer structure with smaller wavelength and optimal film thickness. Junyao Shen, Sulei Fu, 2021, in [77] Design and Fabricate 15° Y-X LiNbO_3_/SiO_2_/SiC Multilayer Substrate, Using SH-mode to Suppress Spurious Responses including Rayleigh Mode and Transverse Mode. The manufactured filters have well-balanced properties. The center frequency is 1.28 GHZ, and the 3 dB FBW reaches 16.65%, which is a high-performance surface acoustic wave device.

**Figure 5:**
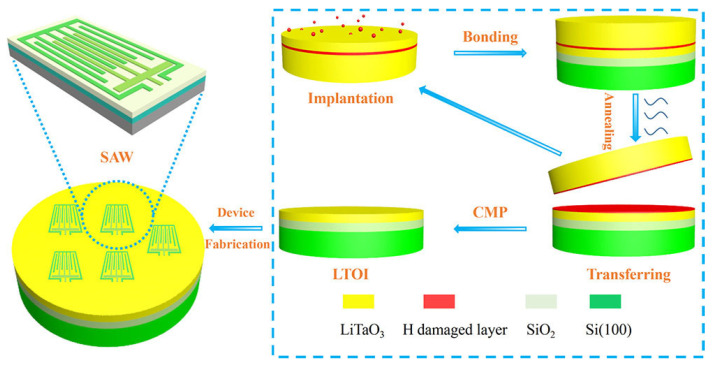
Schematic diagram of the fabrication of surface acoustic wave resonator LTOI [79]. © [2019] IEEE. Reprinted, with permission, from [79].

In 2013, Songbin Gong et al. designed and fabricated the first laterally vibrating microresonator using ion-sliced X-cut LN thin films. High (0002) oriented ZnO piezoelectric films were deposited on high-sonic velocity SiC substrates by conventional photolithography. The frequency is in the 5–7 GHZ range. Has a large K2 value, and Q (146~549) [78]. In 2019, Youquan Yan, using piezoelectric thin films instead of piezoelectric substrates to prepare surface acoustic wave resonators, can obtain higher quality factor (Q) and lower temperature coefficient of frequency (TCF). As shown in Figure 5, (A typical ion cutting process was used to prepare a 42° rotated y-cut LT single-crystal thin film substrate. The process flow for transferring LT thin films) First, the LT sample was implanted at 75 kev H^+^, and through direct wafer bonding, the single-crystal-level LT thin film was Transferred to a Si substrate, post-annealing at 400 °C further improved the crystalline quality, and then a 30 nm damaged layer was removed by an optimized chemical mechanical polishing (CMP) process, resulting in a significant increase in the blistered surface roughness of the LT film from 12 nm. It was reduced to 0.2 nm, and finally a 350 MHZ single-port surface acoustic wave resonator was designed using the prepared LTOI (LiTaO_3_-on-insulator) substrate [79].

Like photonics and other electromagnetic radiation, metamaterials offer an exciting avenue for controlling and manipulating surface acoustic wave propagation, which may lead to new device concepts and paradigms [80]. In 2021, Pouya et al., through simulations and experiments, metamaterial patterning on a lithium niobate substrate allows to control the value of the SAW phase velocity both slower and faster than on an unpatterned substrate. It is demonstrated that phononic metamaterials composed of annular hole resonator arrays can be used to achieve frequency control of surface acoustic wave velocity [81].

Looking forward to the future, thin film technology is the core of the semiconductor industry. The SiO_2_/LiNbO_3_ layer system was studied by picosecond photoacoustic metrology, and the sound velocity of each layer and the film thickness of SiO_2_ were measured, which can be used for the manufacture of surface acoustic wave filters for communication technology. key information, using birefringence and concomitant changes in the detection sensitivity of coherent phonons in LiNbO_3_ layers to infer information about LiNbO_3_ orientation and layer interfaces, which can infer nanoscale film thickness ranges, as well as velocities, while film-dependent Many of the applications of the Picosecond Photoacoustic Metrology of Si require an understanding of the speed of sound and the thickness of the film [Picosecond Photoacoustic Metrology of Si [82]. At the same time filter integration will be the key to cost and scale. How to convert the lumped circuit model into the structural parameters of the acoustic resonator is the key bottleneck for RF circuit designers to use RF circuit design tools to design SAW devices. In 2019, Mei-Hui Chung proposed a method to establish a bridge between the lumped equivalent circuit model of the SAW resonator and the structural parameters [83]. Using a four-layer neural network and BP learning algorithm, the SAW resonator corresponding to the lumped EC was obtained. structure parameters. This method effectively shortens the development time of the SAW filter. There are now sapphire and quartz substrates and IHP and POI on silicon. The advantage of silicon carrier wafers compared to sapphire or quartz is the heat extraction properties of silicon. Silicon conducts heat ~4 times better than sapphire and ~16 times better than quartz [84]. 2021 R. Ruby, in X proposes a new type of lithium lithium tantalate (LT) bonded to a silicon hybrid substrate (SiSAW), which has temperature compensation, good power handling performance, and can eliminate the generation between the LT/Si interface [29]. stray mode. This mode can integrate the required filters on one module, and provides a solution for the sharp rise in the number of RF front-end filters.

## 4. Conclusions

In this era of information explosion, the success of mobile communication plays an irreplaceable role, and the continued success of mobile communication is related to the growing demand for higher transmission data rates. With the standardization of new frequency bands, the designation of new carrier aggregation combinations, the need to increase edge steepness, and generally the need for wider bandwidth and smaller size, this presents a huge challenge for designers. I believe that with the continuous support of various technologies and the integration of filters, the performance of SAW filters will continue to improve, and there will be higher frequency bands. And the proportion of mobile phones and other electronic devices will also increase, bringing more convenience to our lives.

## References

[1] Bauer T., Eggs C., Wagner K., Hagn P. (2015). A Bright Outlook for Acoustic Filtering. IEEE Microw. Mag..

[2] Liu Y., Cai Y., Zhang Y., Tovstopyat A., Liu S., Sun C.L. (2020). Materials, Design, and Characteristics of Bulk Acoustic Wave Resonator: A Review. Micromachines.

[3] Gimenez A., Verdu J., Sanchez P.D. (2018). General Synthesis Methodology for the Design of Acoustic Wave Ladder Filters and Duplexers. IEEE Access.

[4] Fattinger G.G., Volatier A., Al-Joumayly M., Yusuf Y., Aigner R., Khlat N., Granger-Jones M. Carrier aggregation and its challenges—Or: The golden age for acoustic filters. Proceedings of the 2016 IEEE MTT-S International Microwave Symposium (IMS).

[5] Ruppel C.C.W. (2017). Acoustic Wave Filter Technology–A Review. IEEE Trans. Ultrason. Ferroelectr. Freq. Control..

[6] Delsing P., Cleland A.N., Schuetz M.J.A., Knorzer J., Giedke G., Cirac J.I., Srinivasan K., Wu M., Balram K.C., Bauerle C. (2019). The 2019 surface acoustic waves roadmap. J. Phys. D Appl. Phys..

[7] Rayleigh L. (1885). On waves propagated along the plane surface of an elastic solid. Proc. Lond. Math. Soc..

[8] Xiaojuan F., Yi H., Bingjun W., Shuwen C. (2010). Research on dynamic response of single pile to Rayleigh wave in saturated soil based on Winkler model. Chin. J. Appl. Mech..

[9] White R.M., Voltmer F.W. (1965). Direct piezoelectric coupling to surface elastic waves. Appl. Phys. Lett..

[10] Arnau A. (2004). Piezoelectric Transducers and Applications.

[11] Drafts B. (2001). Acoustic wave technology sensors. IEEE Trans. Microw. Theory Tech..

[12] Malocha D.C. Evolution of the SAW transducer for communication systems. Proceedings of the IEEE Ultrasonics Symposium.

[13] Li Q., Fu S.L., Song C., Wang G.Y., Zeng F., Pan F. (2018). Improved resistance to electromigration and acoustomigration of Al interdigital transducers by Ni underlayer. Rare Met..

[14] Huang Y., Bao J., Tang G., Wang Y., Omori T., Hashimoto K.-Y. (2017). Multimode filter composed of single-mode surface acoustic wave/bulk acoustic wave resonators. Jpn. J. Appl. Phys..

[15] Tian Y.H., Wang L.T., Wang Y.Y., Li Y., Wu H.X., Qian L.R., Li H.L., Wu J.H., Wang J. (2021). Research in Nonlinearity of Surface Acoustic Wave Devices. Micromachines.

[16] Tobolka G., Faber W., Albrecht G., Pilz D. High Volume TV-IF Filter Design, Fabrication, and Applications. Proceedings of the IEEE 1984 Ultrasonics Symposium.

[17] Maskay A., Hummels D.M., da Cunha M.P. (2019). In-Phase and Quadrature Analysis for Amplitude and Frequency Modulations Due to Vibrations on a Surface-Acoustic-Wave Resonator. IEEE Trans. Ultrason. Ferroelectr. Freq. Control.

[18] Morita T., Watanabe Y., Tanaka M., Nakazawa Y. Wideband low loss double mode SAW filters. Proceedings of the IEEE 1992 Ultrasonics Symposium Proceedings.

[19] Beaudin S., Damphousse S., Cameron T. Shoulder Suppressing Technique for dual mode SAW resonators. Proceedings of the 1999 IEEE Ultrasonics Symposium Proceedings, International Symposium (Cat. No. 99CH37027).

[20] Kinsman R.G. A history of crystal filters. Proceedings of the 1998 IEEE International Frequency Control Symposium (Cat. No. 98CH36165).

[21] Cai T., Chen C., Zhang X., Lin F.J., Zhang H.L. (2020). A Hybrid Transmission-Line/SAW-Resonator Analog Signal-Interference Dual-Band Bandpass Filter. IEEE Microw. Wirel. Compon. Lett..

[22] Zhang R.Q., Abdelfattah M., Yang L., Gomez-Garcia R., Peroulis D. (2020). A Hybrid Low-Cost Bandpass Filter with SAW Resonators and External Lumped Inductors Using a Dual-Coupling Scheme. IEEE Trans. Microw. Theory Tech..

[23] Miura M., Matsuda T., Satoh Y., Ueda M., Ikata O., Ebata Y., Takagi H. Temperature compensated LiTaO_3_ sapphire bonded SAW substrate with low loss and high coupling factor suitable for US-PCS application. Proceedings of the IEEE Ultrasonics Symposium.

[24] Rizzato S., Scigliuzzo M., Chiriacò M.S., Scarlino P., Monteduro A.G., Maruccio C., Tasco V., Maruccio G. (2017). Excitation and time resolved spectroscopy of SAW harmonics up to GHz regime in photolithographed GaAs devices. J. Micromech. Microeng..

[25] Kobayashi H., Tohyama K., Hori Y., Iwasaki Y., Suzuki K. A study on temperature-compensated hybrid substrates for surface acoustic wave filters. Proceedings of the 2010 IEEE International Ultrasonics Symposium.

[26] Liu Y.H., Liu J.S., Wang Y.L., Lam C.S. (2019). A Novel Structure to Suppress Transverse Modes in Radio Frequency TC-SAW Resonators and Filters. IEEE Microw. Wirel. Compon. Lett..

[27] Hashimoto K., Kadota M., Nakao T., Ueda M., Miura M., Nakamura H., Nakanishi H., Suzuki K. Recent development of temperature compensated SAW Devices. Proceedings of the 2011 IEEE International Ultrasonics Symposium.

[28] Matsuoka N., Li X., Omori T., Hashimoto K.-Y. (2020). Study of loss mechanisms in temperature compensated surface acoustic wave devices based on finite element method analysis using hierarchical cascading technique. Jpn. J. Appl. Phys..

[29] Ruby R., Gilbert S., Lee S.K., Nilchi J., Kim S.W. (2021). Novel Temperature-Compensated, Silicon SAW Design for Filter Integration. IEEE Microw. Wirel. Compon. Lett..

[30] Yamanouchi K., Kotani K., Odagawa H., Cho Y.S. (2000). Theoretical analysis of surface acoustic wave propagation characteristics under strained media and applications for high temperature stable high coupling surface acoustic wave substrates. Jpn. J. Appl. Phys..

[31] Matsuda S., Hara M., Miura M., Matsuda T., Ueda M., Satoh Y., Hashimoto K. (2011). Correlation Between Temperature Coefficient of Elasticity and Fourier Transform Infrared Spectra of Silicon Dioxide Films for Surface Acoustic Wave Devices. IEEE Trans. Ultrason. Ferroelectr. Freq. Control..

[32] Abbott B., Chen A., Daniel T., Gamble K., Kook T., Solal M., Steiner K., Aigner R., Malocha S., Hella C. Temperature compensated saw with high quality factor. Proceedings of the 2017 IEEE International Ultrasonics Symposium (IUS).

[33] Saleh A.N., Attar A.A., Ahmed O.K., Mustafa S.S. (2021). Improving the thermal insulation and mechanical properties of concrete using Nano-SiO_2_. Results Eng..

[34] Lee S.A., Yang J.W., Choi S., Jang H.W. (2021). Nanoscale electrodeposition: Dimension control and 3D conformality. Exploration.

[35] Liu Y., Shi B., Liang X.J. (2021). Exploration: Explore a better future with advanced science and technology. Exploration.

[36] Yujun Z., Fanxiu L., Jianjun Z., Liangxian C. (2008). Technique of preparing diamond films on poly-substrate by DC-arc plasma jet CVD for surface acoustic wave devices. J. Univ. Sci. Technol. Beijing.

[37] Xue P.J., Liu S.L., Bian J.J. (2021). Effects of polymorphic form and particle size of SiO_2_ fillers on the properties of SiO_2_–PEEK composites. J. Adv. Dielectr..

[38] Balani S.B., Ghaffar S.H., Chougan M., Pei E.J., Sahin E. (2021). Processes and materials used for direct writing technologies: A review. Results Eng..

[39] Goto R., Nakamura H., Hashimoto K. (2019). The modeling of the transverse mode in TC-SAW using SiO_2_/LiNbO_3_ structure. Jpn. J. Appl. Phys..

[40] Kadota M., Nakao T., Taniguchi N., Takata E., Mimura M., Nishiyama K., Hada T., Komura T. (2005). Surface acoustic wave duplexer for US personal communication services with good temperature characteristics. Jpn. J. Appl. Phys..

[41] Kovacs G., Ruile W., Jakob M., Rosler U., Maier E., Knauer U., Zoul H. A SAW duplexer with superior temperature characteristics for US-PCS. Proceedings of the IEEE Ultrasonics Symposium.

[42] Kadota M., Nakao T., Taniguchi N., Takata E., Mimura M., Nishiyama K., Hada T., Komura T. SAW duplexer for PCS in US with excellent temperature stability. Proceedings of the IEEE Symposium on Ultrasonics.

[43] Kadota M., Nakao T., Nishiyama K., Kido S., Kato M., Omote R., Yonekura H., Takada N., Kita R. (2007). Small surface acoustic wave duplexer for wide-band code-division multiple access full-band system having good temperature characteristics. Japanese J. Appl. Phys..

[44] Nakamura H., Nakanishi H., Tsurunari T., Matsunami K., Iwasaki Y., Hashimoto K., Yamaguchi M. (2008). Miniature Surface Acoustic Wave Duplexer Using SiO_2_/Al/LiNbO_3_ Structure for Wide-Band Code-Division Multiple-Access System. Jpn. J. Appl. Phys..

[45] Matsuda S., Hara M., Miura M., Matsuda T., Ueda M., Satoh Y., Hashimoto K. Application of fluorine doped SiO_2_ films for temperature compensated SAW devices. Proceedings of the 2011 IEEE International Ultrasonics Symposium.

[46] Wang Y., Solal M., Kook T., Briot J., Abbott B., Chen A., Daniel T., Malocha S., Qin K., Steiner K. A zero TCF band 13 SAW duplexer. Proceedings of the 2015 IEEE International Ultrasonics Symposium (IUS).

[47] Koskela J., Plessky V., Willemsen B., Turner P., Hammond B., Fenzi N. (2018). Hierarchical Cascading Algorithm for 2-D FEM Simulation of Finite SAW Devices. IEEE Trans. Ultrason. Ferroelectr. Freq. Control..

[48] Solal M., Gallagher M., Tajic A. Full 3D simulation of SAW resonators using hierarchical cascading FEM. Proceedings of the 2017 IEEE International Ultrasonics Symposium (IUS).

[49] Li X., Bao J., Qiu L., Matsuoka N., Omori T., Hashimoto K.-Y. (2019). 3D FEM simulation of SAW resonators using hierarchical cascading technique and general purpose graphic processing unit. Jpn. J. Appl. Phys..

[50] Tsutsumi J., Inoue S., Iwamoto Y., Miura M., Matsuda T., Satoh Y., Nishizawa T., Ueda M., Ikata O. A miniaturized 3 × 3-mm SAW antenna duplexer for the US-PCS band with temperature-compensated LiTaO_3_ sapphire substrate. Proceedings of the IEEE Ultrasonics Symposium.

[51] Kawachi O., Taniguchi N., Tajima M., Nishizawa T. A study of optimum material for SAW bonded wafer. Proceedings of the 2012 IEEE International Ultrasonics Symposium.

[52] Geshi K., Teraoka K., Kinoshita S., Nakayama M., Imagawa Y., Nakayama S., Hashimoto K., Tanaka S., Totsu K., Takagi H. Wafer bonding of polycrystalline spinel with LiNbO_3_/LiTaO_3_ for temperature compensation of RF surface acoustic wave devices. Proceedings of the 2012 IEEE International Ultrasonics Symposium.

[53] Zhang S., Lu R., Zhou H., Link S., Yang Y., Li Z., Huang K., Ou X., Gong S. (2020). Surface Acoustic Wave Devices Using Lithium Niobate on Silicon Carbide. IEEE Trans. Microw. Theory Tech..

[54] Su R., Fu S., Shen J., Lu Z., Xu H., Yang M., Zeng F., Song C., Wang W., Pan F. (2021). Power Durability Enhancement and Failure Analysis of TC-SAW Filter with Ti/Cu/Ti/Cu/Ti Electrodes. IEEE Trans. Device Mater. Reliab..

[55] Ballandras S., Courjon E., Bernard F., Laroche T., Clairet A., Radu I., Huyet I., Drouin A., Butaud E. New generation of SAW devices on advanced engineered substrates combining piezoelectric single crystals and Silicon. Proceedings of the 2019 Joint Conference of the IEEE International Frequency Control Symposium and European Frequency and Time Forum (EFTF/IFC).

[56] Tang I.T., Chen H.J., Hwang W.C., Wang Y.C., Houng M.P., Wang Y.H. (2004). Applications of piezoelectric ZnO film deposited on diamond-like carbon coated onto Si substrate under fabricated diamond SAW filter. J. Cryst. Growth.

[57] Assouar M., Elmazria O., Rioboo R.J., Sarry F., Alnot P. (2000). Modelling of SAW filter based on ZnO/diamond/Si layered structure including velocity dispersion. Appl. Surf. Sci..

[58] Georgel V., Verjus F., van Grunsven E.C.E., Poulichet P., Lissorgues G., Pellet C., Chamaly S., Bourouina T. (2008). A SAW filter integrated on a silicon passive substrate used for system in package. Sens. Actuators A Phys..

[59] Takai T., Iwamoto H., Takamine Y., Yamazaki H., Fuyutsume T., Kyoya H., Nakao T., Kando H., Hiramoto M., Toi T. Incredible high performance SAW resonator on novel multi-layerd substrate. Proceedings of the 2016 IEEE International Ultrasonics Symposium (IUS).

[60] Takai T., Iwamoto H., Takamine Y., Wada T., Hiramoto M., Koshino M., Nakajima N. Investigations on design technologies for SAW quadplexer with narrow duplex gap. Proceedings of the 2016 IEEE MTT-S International Microwave Symposium (IMS).

[61] Takai T., Iwamoto H., Takamine Y., Fuyutsume T., Nakao T., Hiramoto M., Toi T., Koshino M.I.H.P. SAW technology and its application to microacoustic components (Invited). Proceedings of the 2017 IEEE International Ultrasonics Symposium (IUS).

[62] Takai T., Iwamoto H., Takamine Y., Yamazaki H., Fuyutsume T., Kyoya H., Nakao T., Kando H., Hiramoto M., Toi T. (2017). High-Performance SAW Resonator on New Multilayered Substrate Using LiTaO_3_ Crystal. IEEE Trans. Ultrason. Ferroelectr. Freq. Control.

[63] Takamine Y., Takai T., Iwamoto H., Nakao T., Koshino M. A Novel 3.5 GHz Low-Loss Bandpass Filter Using I.H.P. SAW Resonators. Proceedings of the 2018 Asia-Pacific Microwave Conference (APMC).

[64] Takai T., Iwamoto H., Takamine Y., Fuyutsume T., Nakao T., Hiramoto M., Toi T., Koshino M. (2019). High-Performance SAW Resonator with Simplified LiTaO_3_/SiO_2_ Double Layer Structure on Si Substrate. IEEE Trans. Ultrason. Ferroelectr. Freq. Control.

[65] Kadota M., Tanaka S. Improved quality factor of hetero acoustic layer (HAL) SAW resonator combining LiTaO_3_ thin plate and quartz substrate. Proceedings of the 2017 IEEE International Ultrasonics Symposium (IUS).

[66] Kadota M., Yunoki Y., Shimatsu T., Uomot M., Tanaka S. Near-Zero TCF of HAL SAW Resonator with LiTaO_3_-on-Quartz Structure. Proceedings of the 2018 IEEE International Frequency Control Symposium (IFCS).

[67] Kadota M., Ishii Y., Tanaka S. (2020). A spurious-free, steep band rejection filter using a LiTaO3/quartz heteroacoustic layer surface acoustic wave resonator. Jpn. J. Appl. Phys..

[68] Kimura T., Daimon K., Ogami T., Kadota M. (2013). S0Mode Lamb Wave Resonators Using LiNbO_3_ Thin Plate on Acoustic Multilayer Reflector. Jpn. J. Appl. Phys..

[69] Kimura T., Kishimoto Y., Omura M., Hashimoto K. (2018). 3.5 GHz longitudinal leaky surface acoustic wave resonator using a multilayered waveguide structure for high acoustic energy confinement. Jpn. J. Appl. Phys..

[70] Kimura T., Omura M., Kishimoto Y., Hashimoto K.Y. Applicability Investigation of SAW Devices in the 3 to 5 GHz range. Proceedings of the 2018 IEEE/MTT-S International Microwave Symposium—IMS.

[71] Suzuki M., Sawada N., Kakio S. (2019). Analysis of longitudinal leaky surface acoustic wave propagation characteristics on a piezoelectric ScAlN layer/sapphire or quartz substrate. Jpn. J. Appl. Phys..

[72] Tsutsumi J., Matsuda T., Ikata O., Satoh Y. (2000). A novel reflector-filter using a SAW waveguide directional coupler. IEEE Trans. Ultrason. Ferroelectr. Freq. Control.

[73] Tsutsumi J., Ikata O., Satoh Y. (2001). Experimental Studies on Acoustic Energy Loss in Surface Acoustic Wave Filters Using Grating Waveguides. Jpn. J. Appl. Phys..

[74] Li Q., Fu S.L., Wang R., Song C., Zeng F., Pan F. (2018). Enhanced power durability of surface acoustic wave filter with Al/Ti/Cu/Ti electrodes. J. Alloy. Compd..

[75] Li Q., Fu S.L., Lu Z.T., Qian L.R., Wang R., Chen T.J., Song C., Zeng F., Wang W.B., Pan F. (2019). Behavior of Al/Cu/Ti electrodes in surface acoustic wave filter at high power. Curr. Appl. Phys..

[76] Hsu T.H., Tseng K.J., Li M.H. (2020). Large Coupling Acoustic Wave Resonators Based on LiNbO_3_/SiO_2_/Si Functional Substrate. IEEE Electron Device Lett..

[77] Shen J.Y., Fu S.L., Su R.X., Xu H.P., Lu Z.T., Xu Z.B., Luo J.T., Zeng F., Song C., Wang W.B.A. (2021). High-Performance Surface Acoustic Wave Devices Using LiNbO_3_/SiO_2_/SiC Multilayered Substrates. IEEE Trans. Microw. Theory Tech..

[78] Gong S.B., Piazza G. (2013). Design and Analysis of Lithium-Niobate-Based High Electromechanical Coupling RF-MEMS Resonators for Wideband Filtering. IEEE Trans. Microw. Theory Tech..

[79] Yan Y.Q., Huang K., Zhou H.Y., Zhao X.M., Li W.Q., Li Z.X., Yi A.L., Huang H., Lin J.J., Zhang S.B. (2019). Wafer-Scale Fabrication of 42 degrees Rotated Y-Cut LiTaO_3_-on-Insulator (LTOI) Substrate for a SAW Resonator. ACS Appl. Electron. Mater..

[80] Zeighami F., Palermo A., Marzani A. (2021). Rayleigh waves in locally resonant metamaterials. Int. J. Mech. Sci..

[81] Pouya C., Nash G.R. (2021). Sub-and supersonic elastic waves in an annular hole phononic metamaterial. Commun. Mater..

[82] Brick D., Emre E., Grossmann M., Dekorsy T., Hettich M. (2017). Picosecond Photoacoustic Metrology of SiO_2_ and LiNbO_3_ Layer Systems Used for High Frequency Surface-Acoustic-Wave Filters. Appl. Sci..

[83] Chung M.H., Huang H.C., Hwang R.C., Huang P.F., Wang S.M.T. (2019). Synthesis of Ladder-type Radio Frequency Surface Acoustic Wave Filter Based on Lumped Circuit Model by Using Neural Network. Sens. Mater..

[84] Yang S., Ai Y.J., Cheng Z., Zhang L.A., Jia L.F., Dong B.Y., Zhang B.H., Wang J.X., Zhang Y. (2019). Method of the out-of-band rejection improvement of the AlN based surface acoustic wave filters. Ultrasonics.

